# Draft Genome Sequence of *Clostridium difficile* Strain IT1118, an Epidemic Isolate Belonging to the Emerging PCR Ribotype 018

**DOI:** 10.1128/genomeA.00717-16

**Published:** 2016-07-21

**Authors:** François Wasels, Fabrizio Barbanti, Patrizia Spigaglia

**Affiliations:** Department of Infectious, Parasitic and Immune-Mediated Diseases, Istituto Superiore di Sanità, Rome, Italy

## Abstract

*Clostridium difficile* PCR ribotype 018 has emerged in Italy, South Korea, and Japan, causing severe infections and outbreaks. In this study, we sequenced the genome of IT1118, an Italian clinical isolate, to clarify the molecular features contributing to the success of this epidemic type.

## GENOME ANNOUNCEMENT

*Clostridium difficile* is the main causative agent of nosocomial diarrhea. Besides hypervirulent PCR ribotypes (RTs), other epidemic RTs are currently a cause of concern (1, 2). In particular, RT018 has been detected in Italy and, more recently, in South Korea and Japan as a main cause of infections and outbreaks (3–5). RT018 isolates show resistance to several antibiotics and a transmission index 10-fold higher compared to that of RT078 strains (3, 6). Significant risk factors for complicated infections by RT018 are age ≥65 years, pulmonary comorbidity, and use of fluoroquinolones (2). For these reasons, the genome of the Italian strain IT1118, isolated during an outbreak, was analyzed to investigate features that may affect virulence.

Genomic DNA was sequenced using the HiSeq 2000 platform (GATC, Konstanz, Germany) in 50-bp single-read mode. A total of 18,123,358 reads were assembled into 256 contigs (>200 bp) using the Velvet assembler (7), with a total size of 4,238,925 bp and providing 218× coverage. The average contig length was 16,558 bp, with the largest contig being 178,925 bp. Gene prediction was performed using Glimmer version 3 (8), and contigs were mapped against the reference strain *C. difficile* 630 (RT012) (GenBank no. AM180355) using Geneious (9).

Mutations C245T in *gyrA* and G1514A in *rpoB*, found in the majority of European *C. difficile* isolates resistant to fluoroquinolones and rifampins (10–12), respectively, were detected in IT1118. Although IT1118 showed resistance to both erythromycin and clindamycin, neither resistance determinants nor mutations in the ribosomal proteins genes were observed.

The temporal activation of the *sigK* gene, involved in *C. difficile* sporulation, is regulated by the excision of a *sigK* intervening (skin) element (13). Results for the skin element of IT1118 (57 kb) were very different from those of 630 or M120 (RT078), but it had 99.9% sequence identity to that of strain BI-9 (GenBank no. FN668944), which belongs to the long-term problematic epidemic RT001. Diversity in the skin elements could differently affect strain sporulation and consequent transmissibility, although this hypothesis needs to be confirmed.

The surface layer (SL) of *C. difficile* is involved in pathogen-host interactions. Twelve different SL cassettes (containing the *slp*A, *cwp*66, and *secA* genes) have been identified (14). The 9.7-kb SL cassette of IT118 showed 99.9% identity with the cassette 6 (14). Competitive assays *in vivo* suggest that strains with cassette 6 have an advantage in intestinal colonization compared to strains with other SL cassettes (15).

No significant differences in the pathogenicity locus, containing the genes encoding for the toxins A and B and their regulators, were observed between IT1118 and 630 (99.9% of identity). The sequence of the locus for the binary toxin, an additional toxin found in several RTs, was incomplete in both IT1118 and 630.

The genetic characteristics observed in IT1118 show this strain’s high capability to survive and propagate, giving a first explanation for the successful spreading of this *C. difficile* type.

### Nucleotide sequence accession numbers.

This genome sequence has been deposited in DDBJ/EMBL/GenBank under the accession number FAXM00000000. The version described in this paper is the first version, FAXM01000000.
